# DNA methylation loci in placenta associated with birthweight and expression of genes relevant for early development and adult diseases

**DOI:** 10.1186/s13148-020-00873-x

**Published:** 2020-06-03

**Authors:** Fasil Tekola-Ayele, Xuehuo Zeng, Marion Ouidir, Tsegaselassie Workalemahu, Cuilin Zhang, Fabien Delahaye, Ronald Wapner

**Affiliations:** 1 Epidemiology Branch, Division of Intramural Population Health Research, Eunice Kennedy Shriver National Institute of Child Health and Human Development, National Institutes of Health, 6710B Rockledge Dr, room 3204, Bethesda, MD 20892 USA; 2 Division of Intramural Population Health Research, Eunice Kennedy Shriver National Institute of Child Health and Human Development, National Institutes of Health, Bethesda, MD USA; 3 Department of Genetics, Albert Einstein College of Medicine, Bronx, New York, USA; 4 UMR 1283, Institut Pasteur de Lille, Lille, France; 5 Department of Obstetrics and Gynecology, Columbia University, New York, NY USA

**Keywords:** Placenta, Birthweight, Fetal growth, DNA methylation, Transcriptomics, Expression quantitative trait methylation (eQTM), Developmental origins of health and disease (DOHaD)

## Abstract

**Background:**

Birthweight marks an important milestone of health across the lifespan, including cardiometabolic disease risk in later life. The placenta, a transient organ at the maternal-fetal interface, regulates fetal growth. Identifying genetic loci where DNA methylation in placenta is associated with birthweight can unravel genomic pathways that are dysregulated in aberrant fetal growth and cardiometabolic diseases in later life.

**Results:**

We performed placental epigenome-wide association study (EWAS) of birthweight in an ethnic diverse cohort of pregnant women (*n* = 301). Methylation at 15 cytosine-(phosphate)-guanine sites (CpGs) was associated with birthweight (false discovery rate (FDR) < 0.05). Methylation at four (26.7%) CpG sites was associated with placental transcript levels of 15 genes (FDR < 0.05), including genes known to be associated with adult lipid traits, inflammation and oxidative stress. Increased methylation at cg06155341 was associated with higher birthweight and lower *FOSL1* expression, and lower *FOSL1* expression was correlated with higher birthweight. Given the role of the FOSL1 transcription factor in regulating developmental processes at the maternal-fetal interface, epigenetic mechanisms at this locus may regulate fetal development. We demonstrated trans-tissue portability of methylation at four genes (*MLLT1*, *PDE9A*, *ASAP2*, and *SLC20A2*) implicated in birthweight by a previous study in cord blood. We also found that methylation changes known to be related to maternal underweight, preeclampsia and adult type 2 diabetes were associated with lower birthweight in placenta.

**Conclusion:**

We identified novel placental DNA methylation changes associated with birthweight. Placental epigenetic mechanisms may underlie dysregulated fetal development and early origins of adult cardiometabolic diseases.

**Clinical trial registration:**

ClinicalTrials.gov, NCT00912132

## Background

Birthweight is a complex multifactorial trait relevant for health and disease in childhood and adulthood. Abnormal birthweight has been consistently associated with infant mortality and morbidity, childhood obesity, and cardiometabolic diseases in adulthood [1–3]. Complex interplays between genetic and environmental influences play important roles in regulating fetal growth and development [4]. Epigenetic mechanisms such as DNA methylation may represent the regulatory links between genetic and environmental influences on birthweight [5]. Identifying DNA methylation loci that are associated with birthweight can give clues to detect molecular biomarkers of aberrant fetal growth and cardiometabolic diseases in later life. The placenta is known for its prominent relevance in fetal growth and development due to its roles in nutrient transport, immune regulation, and hormonal functions [6]. Compromised placental function has often been associated with aberrant fetal growth [7] and risk of cardiometabolic diseases in later life [8].

DNA methylation in placenta is critical for placental development and regulation of trophoblast invasion, factors important for fetal growth outcomes [9, 10]. The DNA methylation profile of the placenta undergoes dynamic changes [11–13], potentially due to cumulative environmental exposures, adaptive changes in placental cell composition, and genetic regulation [13]. DNA methylation in placenta regulates gene expression and cellular function and may be one mechanism by which environmental exposures and genetic variation influence fetal growth. Therefore, DNA methylation loci associated with birthweight can point to molecular signatures of the intrauterine environment and may lend insights to understand the links between fetal growth and cardiometabolic diseases in later life. Epigenome-wide association studies (EWASs) have identified several cord blood DNA methylation cytosine-phosphate-guanine sites (CpGs) associated with birthweight [14–18]. Although cord blood is a more accessible tissue for genomic studies, the placenta may be a more biologically relevant tissue for studying the epigenetic regulation of fetal growth and development. Previous studies in placenta reported that birthweight status is significantly associated with long interspersed nuclear elements (LINE)-1 methylation in placenta [19, 20], global placental DNA methylation [21], and methylation levels at selected candidate gene regions [22–25] and at CpGs assayed using the HumanMethylation27 BeadChip array [26, 27]. An EWAS of birthweight using placental samples profiled using more recently developed dense DNA methylation arrays is warranted to unravel novel genetic loci.

In this study, we examined the association between birthweight and epigenome-wide DNA methylation in placental samples obtained from participants of the *Eunice Kennedy Shriver* National Institute of Child Health and Human Development (NICHD) Fetal Growth Studies–Singletons cohort [28, 29]. For CpGs found to be significantly associated with birthweight in our analysis, we investigated the associations between methylation levels at the CpGs and expression of the proximal genes in placenta. We also tested for correlations between placental expression of those genes and birthweight. Pathway analyses, functional annotations, and genetic variants associated with DNA methylation were explored to understand the biological context that may explain the relationship between the differentially methylated CpGs and birthweight. The placenta may harbor the epigenetic links between maternal cardiometabolic status, fetal growth and risk of cardiometabolic diseases in later life. Therefore, for CpGs in offspring tissues known to be related to maternal perinatal cardiometabolic traits and CpGs in adult tissues known to be related to cardiometabolic diseases, we tested whether methylation in placenta was associated with birthweight. Our findings of novel genomic loci associated with birthweight and overlaps with adult cardiometabolic diseases lend insights into the pathophysiology of fetal growth and potential molecular targets to prevent cardiometabolic diseases in later life.

## Results

### Data set

We used DNA methylation data measured using Illumina’s Infinium Human Methylation450 Beadchip (Illumina Inc., San Diego, CA) on placentas obtained at delivery from 301 pregnant women who participated in the NICHD Fetal Growth Studies–Singletons [28, 29]. The self-reported race/ethnic profiles of the 301 women were 102 Hispanics, 77 Whites, 72 Blacks, and 50 Asians. None of the pregnant women smoked cigarettes in the past 6 months prior to conception. The mean (± standard deviation (SD)) birthweight and gestational age at delivery were 3165 (± 451) g and 39.5 (± 1.1) weeks, respectively. Detailed characteristics of participants of the NICHD Fetal Growth Studies and the 301 women and neonates included in the present study have been described previously [28, 29]. The major characteristics of the 301 women were similar to those in the overall NICHD Fetal Growth Studies cohort, except pre-pregnancy body mass index (Additional Table 1). Gene expression profile of a subset of the placental samples (*n* = 80) was obtained from RNA sequencing performed on the Illumina HiSeq2000 system. The self-reported race/ethnic profiles of the 80 women were 28 Hispanics, 24 Whites, 20 Blacks, and 8 Asians. Both DNA methylation and gene expression profiles were available in a total of 75 placental samples [29].

### Differentially methylated CpGs associated with birthweight

**Fig. 1:**
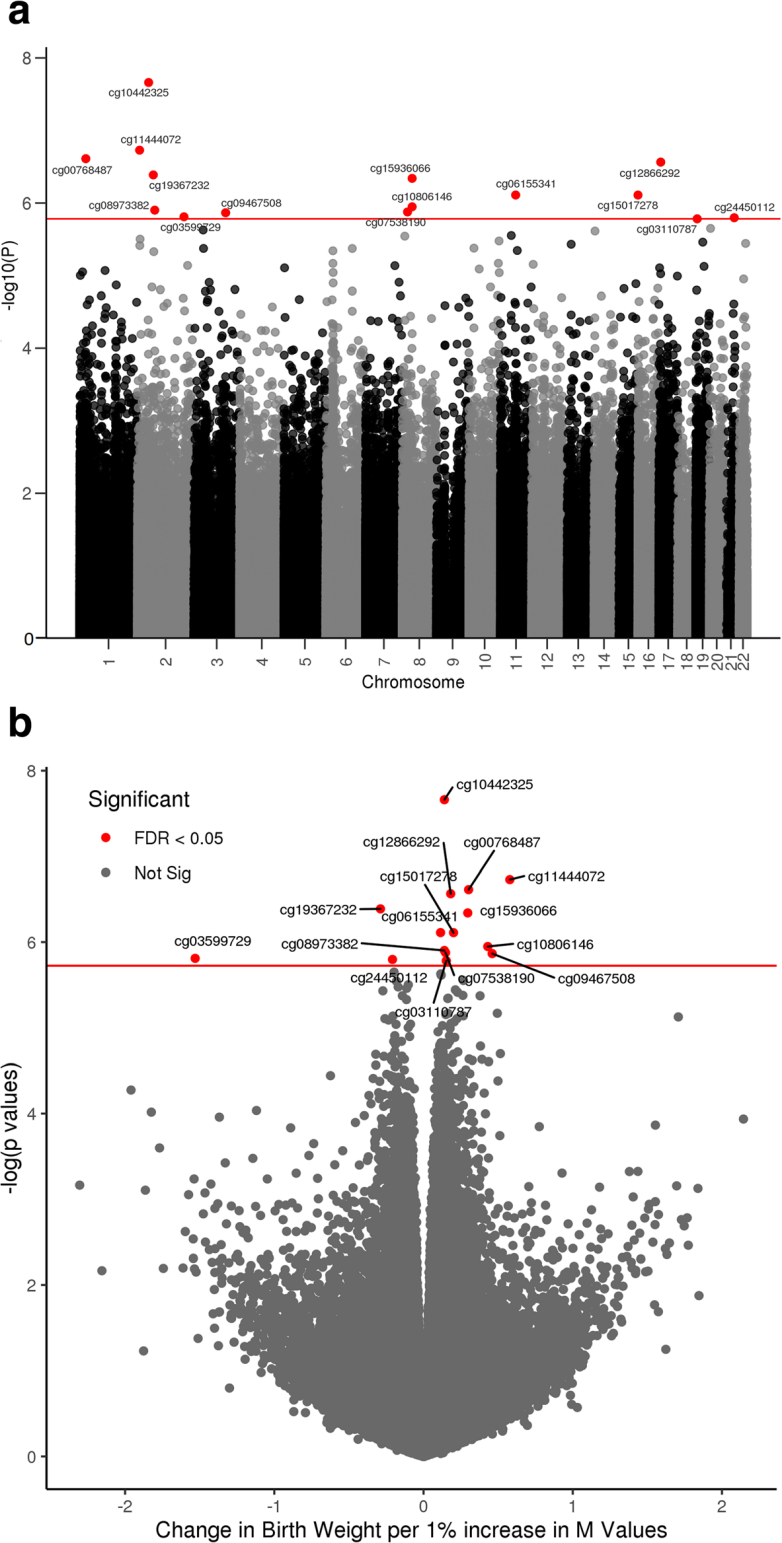
Distributions of associations between CpG sites in placenta and birthweight. **a** Manhattan plot. **b** Volcano plot. Horizontal red line marks FDR 0.05; red dots denote CpGs significantly associated with birthweight

We performed EWAS to identify CpGs significantly associated with birthweight using linear regression models adjusted for neonatal sex, gestational age at delivery, race/ethnicity, five methylation plate representing batch of the 96-well plate on which samples were assigned, top 3 methylation principal components (PCs), top 10 genotype-based PCs representing population stratification due to genetic ancestry, and putative cell mixtures derived from components of surrogate variable analysis (SVA) to account for confounding by cell-type composition [30]. We found that methylation at 15 CpGs was associated with birthweight at a false discovery rate (FDR) of 0.05. The changes in methylation beta values associated with 10 g higher birthweight were found to be highest for cg10806146 (*SLC20A2*) (*β* = 0.04%, 95% CI 0.02% to 0.06%), cg15936066 (*SLC20A2*) (*β* = 0.03%, 95% CI 0.01% to 0.04%), and cg09467508 (intergenic) (*β* = 0.03%, 95% CI 0.01% to 0.04%). Out of the 15 CpGs, 12 (80%) exhibited increased DNA methylation with higher birthweight. Ten CpGs were located in the gene body (*UBXN11*, *ASAP2*, *TMEM107*, *PPP3R1*, *CNIH2*, *MLLT1*, *C2orf60*, *C8orf58, LBX2*, and *PDE9A*), three were within the 5′ untranslated region annotating two genes (*SLC20A2* and *MPG*), and 2 were in intergenic regions (Fig. 1, Table 1). None of the 15 CpGs was associated with gestational age at delivery (Additional Table 2).

**Table 1:** Placental DNA methylation CpG sites associated with birthweight

CpG site	Chr	Position	Gene	Relation to island	Gene group	Coeff (95% CI)	***p*** value	FDR ***p*** value
cg10442325	2	48401950	Intergenic	OpenSea		0.017 (0.009, 0.024)	2.18E− 08	9.0E− 03
cg00768487	1	26611311	*UBXN11*	OpenSea	Body	0.020 (0.006, 0.034)	2.44E− 07	2.8E− 02
cg11444072	2	9427798	*ASAP2*	OpenSea	Body	0.019 (0.005, 0.033)	1.86E− 07	2.8E− 02
cg12866292	17	8079312	*TMEM107*	N_Shore	Body	0.010 (0.004, 0.016)	2.72E− 07	2.8E− 02
cg15936066	8	42356637	*SLC20A2*	OpenSea	5′UTR	0.027 (0.012, 0.041)	4.56E− 07	3.1E− 02
cg19367232	2	68478649	*PPP3R1*	N_Shore	Body	− 0.007 (− 0.010, − 0.004)	4.09E− 07	3.1E− 02
cg06155341	11	66048759	*CNIH2*	N_Shore	Body	0.009 (0.003, 0.014)	7.74E− 07	4.0E− 02
cg15017278	16	127449	*MPG*	Island	5′UTR	0.012 (0.002, 0.022)	7.75E− 07	4.0E− 02
cg03110787	19	6217641	*MLLT1*	S_Shelf	Body	0.012 (0.004, 0.019)	1.65E− 06	4.5E− 02
cg03599729	2	200820225	*C2orf60*	Island	Body	− 0.003 (− 0.004, − 0.001)	1.55E− 06	4.5E− 02
cg07538190	8	22458182	*C8orf58*	S_Shore	Body	0.011 (0.003, 0.019)	1.33E− 06	4.5E− 02
cg08973382	2	74728623	*LBX2*	N_Shore	Body	0.007 (0.002, 0.012)	1.25E− 06	4.5E− 02
cg09467508	3	137486265	Intergenic	N_Shore		0.028 (0.013, 0.044)	1.36E− 06	4.5E− 02
cg10806146	8	42356871	*SLC20A2*	OpenSea	5′UTR	0.041 (0.020, 0.062)	1.13E− 06	4.5E− 02
cg24450112	21	44103949	*PDE9A*	N_Shore	Body	− 0.026 (− 0.039, − 0.013)	1.60E− 06	4.5E− 02

Position: base pair position based on hg19 human reference genome

Relation to island: Shore = 0–2 kb from island, Shelf = 2–4 kb from island, N = upstream (5′) of CpG island, S = downstream (3′) of CpG island

*Coeff* % change in methylation beta value per 10 g increase in birthweight, *FDR* false discovery rate

### Association between DNA methylation at birthweight-associated CpGs and gene expression

We examined whether the 15 CpGs found to be associated with birthweight were related with expression of proximal genes in placenta (i.e., expression quantitative trait methylation loci, eQTMs). We tested this using a linear regression model that included each of the 15 birthweight-associated CpGs and mRNA levels of genes located 500 kb upstream or downstream of the CpGs. There were 272 unique transcripts within 500 kb from the 15 CpGs. We found 15 significant associations (FDR < 0.05) between four CpGs (cg00768487, cg06155341, cg08973382, and cg24450112) and 15 unique gene transcripts (Table 2). For the majority (12/15, 80%) of these eQTMs, higher DNA methylation was associated with decreased gene expression. For the remaining 3 (20%) eQTMs, higher DNA methylation was associated with increased gene expression, consistent with previous evidence that DNA methylation may not always be negatively correlated with gene expression [31]. The CpGs were located upstream relative to the promotor of the associated gene in 8/12 (67%) of the inversely associated eQTMs but were located downstream of the promotor of the associated gene in all positively associated eQTMs. The top associations were between DNA methylation at cg00768487 (that showed increased methylation with higher birthweight in our study) and decreased expression of *SH3BGRL3* and *UBXN11* genes (Table 2).

**Table 2:** Expression of genes in *cis*-region (± 500 kb) of birthweight-associated CpG sites associated with DNA methylation and their correlation with birthweight

CpG site	Gene	Association between gene expression and DNA methylation				Correlation between gene expression and birthweight	
		Coeff	SE	***p*** value	FDR ***p*** value	Pearson ***r***	***p*** value
cg00768487 (chr1)	*SH3BGRL3*	− 0.00055	0.000108	2.30E− 06	5.75E− 05	− 0.145	0.21
	*UBXN11*	− 0.00303	0.000647	1.25E− 05	1.56E− 04	− 0.117	0.31
	RPS6KA1*	− 0.00126	0.000295	5.53E− 05	4.61E− 04	− 0.127	0.27
	CD52*	− 0.0015	0.000407	4.50E− 04	2.81E− 03	− 0.169	0.14
	DHDDS*	− 0.00112	0.000378	4.27E− 03	1.78E− 02	− 0.017	0.88
	*ZNF683*	− 0.0395	0.013312	4.06E− 03	1.78E− 02	− 0.056	0.63
cg08973382 (chr2)	HK2*	− 0.00024	6.43E− 05	2.90E− 04	8.42E− 03	− 0.158	0.17
	SEMA4F*	− 0.00258	0.000747	9.14E− 04	1.32E− 02	− 0.108	0.35
cg06155341 (chr11)	TMEM151A*	− 0.05519	0.012548	3.65E− 05	1.61E− 03	− 0.136	0.24
	CATSPER1*	− 0.00396	0.001153	9.86E− 04	2.17E− 02	− 0.170	0.14
	*KLC2*	0.000382	0.000117	1.62E− 03	2.37E− 02	0.047	0.69
	*CST6*	− 0.00026	8.34E− 05	3.10E− 03	3.41E− 02	− 0.064	0.58
	*RAB1B*	0.00015	5.14E− 05	4.63E− 03	3.62E− 02	0.009	0.93
	FOSL1*	− 0.00391	0.001349	4.93E− 03	3.62E− 02	− 0.248	0.03
cg24450112 (chr21)	*UBASH3A*	0.040597	0.013412	3.41E− 03	4.78E− 02	− 0.209	0.07

*CpG located upstream relative to the promotor (start of gene)

### Correlations between expression of proximal genes and birthweight

**Fig. 2:**
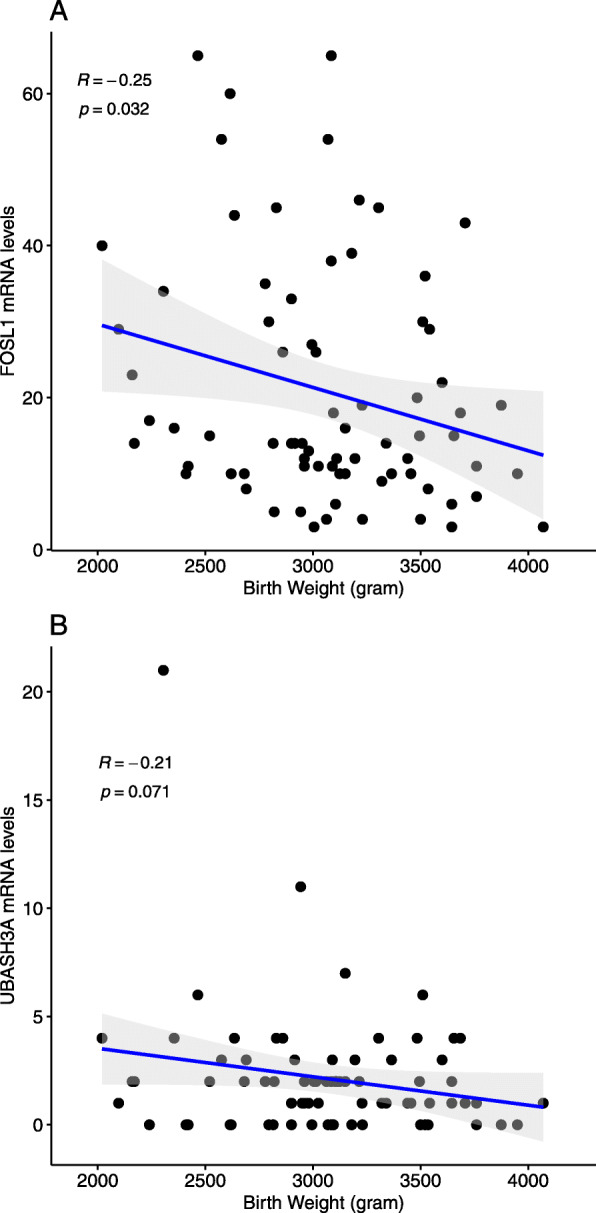
Correlations between birthweight and placental expression of genes associated DNA methylation levels of birthweight-associated CpG sites. **a***FOSL1*. **b***UBASH3A*

**Fig. 3:**
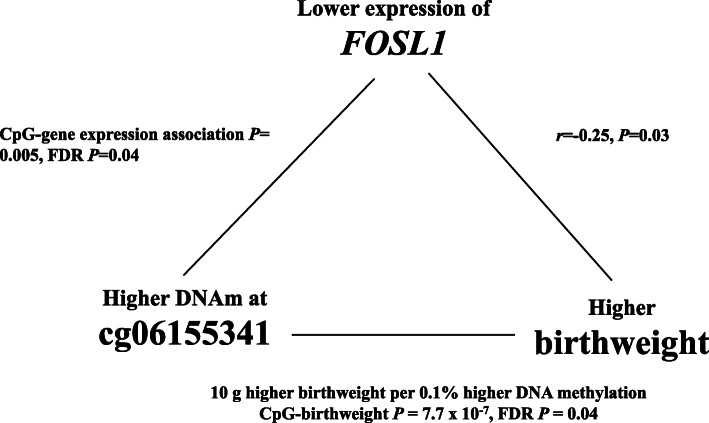
Triangular relationship between DNA methylation at cg06155341, expression of *FOSL1*, and birthweight

Next, we evaluated whether the 15 unique gene transcripts were correlated with birthweight. We found a significant inverse correlation between *FOSL1* expression and birthweight (*r* = − 0.25, *p* = 0.03) (Fig. 2). Notably, increased DNA methylation at cg06155341 was associated with higher birthweight (EWAS finding) and lower *FOSL1* expression (eQTM finding); in parallel, lower *FOSL1* expression was correlated with higher birthweight (gene expression-birthweight correlation finding) (Fig. 3). There was also a tendency for inverse correlations between *UBASH3A* (*r* = − 0.21, *p* = 0.07) and birthweight (Fig. 2).

### Functional annotation and regulatory enrichment

We examined the birthweight-associated CpGs for enrichment in regulatory regions of the genome based on the ENCODE and Roadmap Projects [32]. The birthweight-associated CpGs were enriched (*p* < 0.05) in CpG island shore regions (N-Shore) (*p* = 0.009), chromatin states flanking active TSS (*p* = 0.009), and chromatin states flanking transcription at 5′ and 3′ gene loci (*p* = 0.04) (Additional Table 3).

To further understand the functional relevance of the birthweight-related CpGs, we performed pathway enrichment analysis of genes annotated to the top 100 CpGs using the ingenuity pathway analysis (IPA) tool (QIAGEN Inc., https://www.qiagenbioinformatics.com). The top enriched IPA canonical pathways included cytotoxic T lymphocytes, protein kinase A signaling, mTOR signaling, and virus entry via endocytic pathways. The top enriched diseases and disorders included pathways related to cancer, organismal injury and abnormalities, hematological, immunological and gastrointestinal diseases (Table 3).

**Table 3:** Significantly enriched pathways enriched in genes annotated to top 100 CpGs associated with birthweight

Ingenuity canonical pathways	Molecules	***p*** value	FDR ***p*** value
Assembly of RNA polymerase I complex	POLR1E, POLR1C	1.0E− 03	0.008
CTLA4 signaling in cytotoxic T lymphocytes	AP1G1, FGFR2, PTPN22	1.1E− 02	0.016
DNA methylation and transcriptional repression signaling	CHD4, CHD3	1.1E− 02	0.016
Protein kinase A signaling	FLNB, PPP3R1, PTPRE, PTPN22, ANAPC11, PDE9A	9.0E− 03	0.016
Pyrimidine deoxyribonucleotides de novo biosynthesis I	RRM2, RRM2B	5.0E− 03	0.016
mTOR signaling	FGFR2, PRR5L, ARHGAP8/PRR5-ARHGAP8, PRR5	1.5E− 02	0.018
Virus entry via endocytic pathways	FLNB, AP1G1, FGFR2	1.8E− 02	0.018

We also examined the top 100 CpGs for enrichment of overlap with tissue-specific or cell-type-specific DNase 1 hypersensitivity sites (DHSs) using experimentally derived functional element overlap analysis of regions from EWAS (eFORGE) [33]. The strongest enrichment for tissue-specific DHS was observed in placental cells (*p* = 4.34 × 10^−6^). Significant DHS enrichment was also found in fetal heart, fetal thymus, fetal stomach, and various cells in blood, skin and breast tissues (Additional Table 4).

### *cis*-mQTL annotation in placenta and blood

We assessed genetic determinants of DNA methylation at the 15 birthweight-associated CpGs through a look-up of published resources of *cis*-methylation quantitative loci (*cis*-mQTLs) in placenta [34, 35] and in blood at serial timepoints across the life-course [36]. None of the CpGs overlap with *cis*-mQTLs in placenta, but *cis*-mQTLs have been found in blood for cg03110787 (*MLLT1*), cg12866292 (*TMEM107*), and cg07538190 (*C8orf58*) (Additional Table 5). None of the *cis*-mQTL single nucleotide polymorphisms (SNPs) found in blood has been reported to be associated with birthweight or adult traits in the NHGRI-EBI GWAS catalog of published GWASs [37].

We further assessed whether there are known GWAS loci for birthweight [38] in a 1-Mb window (500 kb up and down stream) of each of the 15 birthweight-associated CpGs. We observed that cg11444072 (*ASAP2*) was located 234 kb and 267 kb, respectively, from the birthweight-associated SNPs rs10495563 and rs11893688 (*ADAM17*), and cg06155341 (*CNIH2*) was located 454 kb from the birthweight-associated SNP rs7102454 (*SNX32*/*EFEMP2*). Absence of *cis*-acting SNPs near the majority of our CpGs shows that the associations are unlikely to be confounded by the effect of common sequence variants on birthweight.

### Phenotypic annotation of birthweight-associated CpGs and genes

**Fig. 4:**
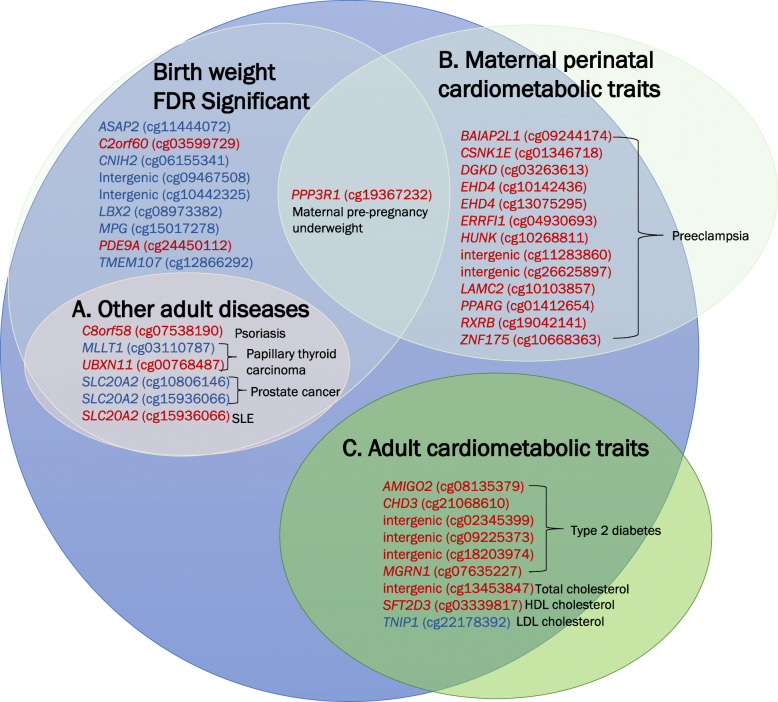
Overlap in CpGs significantly associated with birthweight in placenta and adult traits. **A** Non-cardiometabolic adult diseases. **B** Maternal perinatal cardiometabolic conditions. **C** Cardiometabolic traits in adults. Gene and CpG names in red represent DNA methylation changes associated with increased risk of adult disease as well as lower birthweight. Gene and CpG names in blue represent DNA methylation changes associated with increased risk of adult disease and higher birthweight. SLE stands for systemic lupus erythematosus

Using the EWAS Atlas database [39] and the NHGRI-EBI catalog of published GWASs [37], we assessed whether our birthweight-associated CpGs and annotated genes have been reported to be associated with diseases or traits. We found that cg00768487 (*UBXN11*) and cg03110787 (*MLLT1*) have been associated with papillary thyroid carcinoma [40], cg10806146 (*SLC20A2*) and cg15936066 (*SLC20A2*) with prostate cancer [41], cg15936066 (*SLC20A2*) with systemic lupus erythematosus [42], and cg07538190 (*C8orf58*) with psoriasis [43] (Fig. 4A, Additional Table 6). The *CNIH2* gene is a GWAS locus for gout [44, 45]. Out of the 15 top genes in our study, four genes (*MLLT1*, *PDE9A*, *ASAP2*, and *SLC20A2*) have been mapped to CpGs associated with birthweight in a recent cord blood EWAS meta-analysis [18]. However, the birthweight-associated CpGs annotated to the four genes in cord blood were different from the birthweight-associated placental CpGs we found. The directions of association of the cord blood CpGs were identical with the placental CpGs for *MLLT1* and *PDE9A* but were opposite to the placental CpGs for *ASAP2* and *SLC20A2*.

Next, we examined whether cord blood methylation at CpGs known to be associated with birthweight [14–18] was replicated in placenta. Although no CpG was associated with birthweight at FDR < 0.05, we observed signals of suggestive overlap (FDR = 0.055 to 0.059 in our data) at three CpGs reported to be associated with birthweight (cg01613077 in *RASSF2* (*p* = 2.24 × 10^−6^, FDR =0.055), cg24016995 in *PTPRE* (*p* = 3.3410^−6^, FDR = 0.059), cg03047995 in *FITM1* (*p* = 2.44 × 10^−6^, FDR = 0.055)). At cg01613077 and cg24016995, higher methylation in placenta was associated with higher birthweight in our study consistent with the published study’s findings in cord blood [18]. In contrast, at cg03047995, higher methylation was associated with higher birthweight in our study but with lower birthweight in the published study in cord blood [18].

### Overlap with CpGs previously associated with perinatal maternal cardiometabolic traits

Birthweight is associated with maternal perinatal cardiometabolic status [46]. Therefore, we examined our data to assess whether CpGs in offspring tissues found in previous EWAS using blood samples to be associated with maternal perinatal cardiometabolic traits (including maternal pre-pregnancy BMI (*n* = 1981 CpGs), hypertensive disorders of pregnancy (*n* = 19 CpGs), and preeclampsia (*n* = 84 CpGs); Additional Table 7) overlap with CpGs in placenta associated with birthweight. We found 13 CpGs for which higher DNA methylation in placenta has been related to preeclampsia in previous studies and with lower birthweight in our study (FDR < 0.05). Moreover, at cg19367232 (*PPP3R1*), for which higher DNA methylation in neonatal cord blood has been related to maternal pre-pregnancy underweight in a previous study, higher DNA methylation in placenta was associated with lower birthweight in our study (Fig. 4B, Additional Table 8).

### Overlap with CpGs previously associated with adult cardiometabolic traits

Several studies have also reported that birthweight is associated with offspring risk of cardiometabolic diseases in later life [1–3]. For CpGs known to be associated with childhood/adult cardiometabolic traits in previous EWAS (including myocardial infarction (*n* = 189 CpGs), lipid traits (*n* = 792 CpGs), blood pressure-related traits (*n* = 18 CpGs), type 2 diabetes (*n* = 5672 CpGs), and body mass index (*n* = 1079 CpGs); Additional Table 7), we examined whether DNA methylation at these CpGs in placenta was associated with birthweight. We found six CpGs for which DNA methylation in blood has been associated with adult type 2 diabetes in previous studies and DNA methylation in placenta was associated with birthweight in our study. For all the six CpGs, the methylation change related to increased risk for type 2 diabetes was associated with lower birthweight. Overlapping associations were also found for three CpGs that have been associated with lipid traits in previous studies (Fig. 4C, Additional Table 8).

## Discussion

In this placental EWAS of birthweight, a developmental trait associated with morbidity in childhood and cardiometabolic and cognitive dysfunction in adults [1, 3, 47–50], we identified significant differential methylation at 15 CpG sites. DNA methylation at one third of the birthweight-associated CpG sites was associated with expression of proximal genes in placenta. Notably, the triangular relationships observed between methylation of cg06155341, *FOSL1* gene expression, and birthweight was suggestive of gene expression-mediated influence of DNA methylation on birthweight. We demonstrated that genes annotated to the top-significant CpGs were enriched in pathways including cytotoxic T-lymphocyte antigen-4 (CTLA4), protein kinase A, and mTOR signaling that play key roles in organ development, immune regulation, and cardiometabolic function in humans. Out of the 15 CpGs, methylation at five CpGs in adult blood has been associated with adult chronic diseases [40–43]. In addition, we observed that DNA methylation at CpG sites known to be associated with higher risk of maternal preeclampsia, pre-pregnancy underweight, and adult type 2 diabetes was also significantly associated with lower birthweight in our study. This is consistent with the known inverse correlations observed between these cardiometabolic conditions and birthweight. These findings suggest that placental DNA methylation at loci that play a role in birthweight regulation may be related with maternal perinatal cardiometabolic status and offspring risk of later life chronic diseases.

Previous EWASs have identified several differentially methylated CpGs in cord blood associated with birthweight [14–18]. Four genes (*ASAP2*, *MLLT1*, *PDE9A*, and *SLC20A2*) annotating CpGs associated with birthweight in our study also annotate other CpGs reported to be associated with birthweight in a recent meta-analysis of birthweight EWASs in cord blood [18]. On the one hand, the cross-tissue portability of these genes in relation to birthweight strengthens the evidence for an important biological significance of the genes in fetal growth regulation. None of these four birthweight-associated CpGs were associated with gene expression in placenta in our study or in cord blood in the meta-analysis study [18]. Therefore, it is possible that they may have functional effects other than gene expression, such chromatin stability and alternative splicing which have not been investigated in our study. A recent GWAS of birthweight [38] has also identified a fetal genetic variant in *ASAP2* within 250 kb from the CpGs associated with birthweight in our study, suggesting a potential regulatory relationship between the genetic variant and epigenetic alterations at this locus. On the other hand, the birthweight-associated CpGs annotated to the four genes differ in the two studies. Moreover, for *ASAP2* and *SLC20A2*, the direction of association between birthweight and DNA methylation in placenta was opposite to the direction of association previously seen in cord blood. These differences may be due to possible tissue differences in DNA methylation at CpGs associated with intrauterine influences. It may also be due to complex relationships between genetic variants and DNA methylation at different CpGs on the same gene. Future studies are needed to understand these differences and shed light on mechanisms by which CpGs on the same gene could have directionally opposite associations with birthweight in placenta and cord blood.

Genes annotating CpGs found to be associated with birthweight in our study have functions relevant for fetal growth and placental function. *UBXN11* regulates cellular processes such as protein degradation. Mouse knockout of a member of the UBX family of genes resulted in early embryonic lethality suggesting that the gene is essential for fetal development [51]. An EWAS involving cardiac tissue samples has found differential methylation of a CpG in *UBXN11* associated with dilated cardiomyopathy in adults [52]. *TMEM107* encodes a transmembrane protein that regulates primary cilia, cell surface organelles that respond to environmental cues and play important roles in developmental processes including skeletal formation, neural crest cell formation, and differentiation [53]. *SLC20A2* encodes a protein essential for maternal to fetal phosphate transport, and deficiency of the SLC20A2 protein in mice has been associated with placental vascular defects, increased calcification and fetal growth restriction [54]. The genes for which DNA methylation at birthweight-associated CpG sites exhibited significant association with gene expression in our study are known GWAS loci for adult lipid traits (e.g., *ZDHHC18*) [55], and inflammation/oxidative stress biomarker concentrations (e.g., *ZNF683*) [56].

*FOSL1* (Fos-like antigen 1) encodes a leucin zipper protein involved in the formation of the AP-1 transcription factor complex. The FOS proteins regulate cell proliferation, differentiation and transformation [57]. *FOSL1* has been implicated in bone formation [58] and is a key downstream effector of the PI3K/AKT signaling pathway that regulates placental trophoblast cell invasion [59, 60]. Trophoblast cell invasion is critical for successful placentation and remodeling of the maternal uterine vasculature and can lead to poor fetal growth and other pregnancy complications when disrupted [60]. In vitro experiment in rats has also demonstrated that *FOSL1* regulates trophoblast invasion-vascular remodeling-related genes such as *Sema6d* [59], a member of the semaphorin family of proteins that are involved in signaling and morphogenesis. Further, we found association between DNA methylation at another birthweight-associated CpG site (cg00768487 in *MLLT1*) and *SEMA4F* gene expression, another member of the semaphorin proteins. In all, these findings suggest that the birthweight-associated DNA methylation loci identified in this study are involved in key developmental processes related to epigenetic regulation of the maternal-fetal interface and fetal growth.

Interestingly, ten out of the 15 birthweight-associated CpGs found in the present study have been implicated in gestation duration at delivery by a study that identified more than 5000 CpGs associated in neonatal cord blood with gestational age [61]. Our findings are unlikely to be due to confounding by gestational age at delivery because the model was adjusted for gestational age and none of the ten CpGs were associated with gestational age in sensitivity analysis. At nine CpGs, the direction of association of cord blood methylation with gestational age in the previous study [61] was opposite to the direction of association of placental methylation with birthweight in our study. Therefore, we speculate that the association of higher methylation at these loci with shorter duration of gestation may be related to the increased fetal size, because bigger fetal size at term gestation contributes to labor onset via enhanced mechanical uterine stretch [62] and release of fetal maturation signals [63]. Future studies are needed to understand the role of these loci in the link between fetal size and initiation of parturition.

Maternal perinatal cardiometabolic status has been associated with birthweight [46], and birthweight has been associated with offspring risk of cardiometabolic diseases in later life [1–3]. Understanding the mechanisms that underlie these links is useful to develop early interventions. We found 14 CpGs exhibiting overlapping associations with maternal pre-pregnancy underweight and preeclampsia in previous studies and with lower birthweight in our study. It is therefore possible that DNA methylation changes at these loci may partly explain the relationships of maternal pre-pregnancy underweight and preeclampsia with lower birthweight [64]. We also found six CpGs for which DNA methylation in placenta was related to lower birthweight in the present study and with higher risk of type 2 diabetes in adult blood in previous studies, consistent with the inverse genetic correlations between birthweight and risk of type 2 diabetes [38].

This study has key strengths such as inclusion of racial/ethnic diverse pregnant women and availability of SNP genotype data that helped adjust for genotype PCs to minimize bias due to population structure. The use of multiple data sources and integrated analysis of DNA methylation with transcriptomic data provided insights into potential downstream effects of the identified methylation changes. In depth in silico functional analyses facilitated functional interpretation of the findings and their relevance for adult diseases. Ours is the first study to date to examine the associations of densely profiled genome-wide DNA methylation in placenta, an important tissue in fetal growth, with birthweight.

We acknowledge that there are limitations to our study. We cannot definitively determine whether the altered DNA methylation profiles led to birthweight differences or were a response of the placenta to birthweight differences. To date, there is no reference data for cell proportion in placenta; hence, the altered DNA methylation profiles at the CpGs may be related to unmeasured heterogeneity or maturity of cells in placenta. To counter this limitation, we implemented reference-free adjustment using SVA which adjusts for technical and unmeasured confounding due cell-type proportion variation [65]. Confounding due to unknown factors associated with both placental DNA methylation and birthweight may have influence on our results. Our analysis did not detect substantially large DNA methylation changes associated with birthweight. All placental samples were obtained from deliveries at or near term (mean ± SD gestational age at delivery = 39.5 ± 1.1 weeks); hence, to what extent the findings of this study are generalizable to earlier gestation times remains uncertain. Future studies are needed to determine the functional role of the loci discovered in the present study and larger cohorts can potentially identify additional methylation loci associated with birthweight.

## Conclusions

This first placental EWAS of birthweight identified 15 DNA methylation CpGs significantly associated with birthweight, with several CpGs being associated with expression of nearby genes in placenta. Some of the birthweight-associated loci play key roles in developmental processes and have been associated with adult cardiometabolic diseases. The findings suggest that the placental genes associated with birthweight in our study may be important for understanding the molecular mechanisms linking birthweight to later life cardiometabolic diseases.

## Methods

### Data set and study population

The present study included 312 women who provided placenta samples at delivery as part of the *Eunice Kennedy Shriver* National Institute of Child Health and Human Development (NICHD) Fetal Growth Studies–Singletons. Out of the 312 women, 301 were available for analysis after quality control procedures described below. The NICHD Fetal Growth Studies–Singletons is a prospective longitudinal cohort of 2802 pregnant woman without major pre-existing medical conditions from four self-identified race/ethnic groups (i.e., non-Hispanic White, non-Hispanic Black, Hispanic, and Asian or Pacific Islander) recruited from 12 clinic sites in the USA and followed through delivery [28, 66]. Details about the study design and data collection methods have been previously reported [28, 66]. Gestational age was determined using the date of the last menstrual period and confirmed by ultrasound between 8 weeks to 13 weeks and 6 days of gestation [66]. The study was approved by institutional review boards at NICHD and each of the participating clinical sites. Written informed consent was obtained from all study participants.

### Birthweight measurements

After delivery, neonates underwent standardized anthropometric measurements as previously described [67]. Birthweight was measured in grams using an infant beam-balance or digital scale.

### Placental sample collection and DNA methylation quantifications

At delivery, placentas were acquired by trained research personnel. The placentas were rinsed with sterile saline and pat dried with paper towel, and nonadherent blood clots were removed. The placental membrane and umbilical cord were trimmed before biopsies were taken. Placental biopsies measuring 0.5 cm × 0.5 cm × 0.5 cm were collected directly below the fetal surface of the placenta within one hour of delivery. Samples were placed in RNALater and frozen for molecular analysis. Processing of the placental biopsies was performed at the Columbia University Irving Medical Center and details have been described previously [35]. DNA from placental biopsies was extracted and assayed using Illumina’s Infinium Human Methylation450 Beadchip (Illumina Inc., San Diego, CA). Standard Illumina protocols were followed for background correction, normalization to internal control probes, and quantile normalization. Normalization was performed using the modified Beta MIxture Quantile dilation (BMIQ) method to correct the probe design bias in the Illumina Infinium Human Methylation450 Beadchip and achieve between-sample normalization [68, 69]. The resulting intensity files were processed with Illumina’s GenomeStudio, which generated average betascores (i.e., the fraction of methylated sites per sample by taking the ratio of methylated and unmethylated fluorescent signals at each queried CpG) and detection *p* values. Details of quality control filters applied on the genotype and DNA methylation data have been previously reported [29]. Beta scores with an associated detection *p* values ≥ 0.05 were set to missing. In addition, probes with mean detection *p* value ≥ 0.05 (*n* = 36), cross-reactive (*n* = 24,491), non-autosomal (*n* = 14,589), and CpG sites located within 20 base pairs from known single nucleotide polymorphisms (SNPs) (*n* = 37,360) were removed. After these QC procedures, 409,101 CpGs were included in the EWAS. As recommended by Du et al. [70], beta values for each CpG site (i.e., the fraction of methylated sites per sample by taking the ratio of methylated and unmethylated fluorescent signals) were logit transformed to *M*-value scale.

### Placental genotyping

Placental DNA samples were genotyped using HumanOmni2.5 Beadchips (Illumina Inc., San Diego, CA), followed by initial data processing using Illumina’s Genome Studio, as previously described [35]. SNPs were excluded if they had excessive missing genotype (SNPs with genotype call rate of < 95%), deviated from Hardy-Weinberg equilibrium (*p* value < 0.0001), and had low minor allele frequency (< 0.05). A total of 11 samples showing discrepancies between phenotypic sex and genotypic sex (*n* = 4), that were outliers from the distribution of the samples’ genetic clusters based on multi-dimensional scaling plots (*n* = 6), and with a mismatching sample identifier (*n* = 1) were excluded.

### Placental RNA extraction and quantification

RNA was extracted from 80 placentas using TRIZOL reagent (Invitrogen, MA), and sequenced using the Illumina HiSeq2000 system. The RNA sequence preprocessing procedure has been previously described [35]. Using the RNA-seq reads, the expression of the transcripts were quantified using Salmon [71]. A total of 75 samples with both methylation and RNA-seq data were included in the tests for associations between DNA methylation and gene expression, as well as covariate-adjusted associations between gene expression and birthweight.

### Epigenome-wide association analysis

Epigenome-wide analyses were performed by fitting simple linear regression models on the methylation *M*-values of each CpG as the dependent variable and birthweight as independent variable as implemented in the R/Bioconductor package limma, version 3.24.15. So far, there is no reference for placental cell-type composition; therefore, our model included SVA using the Combat function that has been validated to account for heterogeneity in cell-type composition [30]. Placental genome-wide SNP data were used to estimate 10 genotype-based PCs representing population structure. The R package “prcomp” was used to calculate methylation-based PCs [72]. All analyses adjusted for gestational age at birth (continuous), neonatal sex (male, female), maternal race/ethnicity (Hispanic, Black, White, Asian), five methylation plates representing batch of the 96-well plate on which samples were assigned, top ten genotype-based PCs based on the eigenvectors which represent most of the variance in the genotype data, top three methylation-based PCs, and 20 SVA components that represent putative cell-type composition in our samples. None of the women studied reported smoking in the 6 months prior to pregnancy. Quantile-quantile (QQ) plots of *p* values and the corresponding genomic inflation factor (*λ* = 1.23) are presented in Additional Figure 1. Statistical significance was considered based on FDR of 5%. The differentially methylated CpG sites were mapped to the nearby gene using R/Bioconductor package with a background reference comprising the whole set of genes present in the Illumina 450 k platform. To simplify interpretation, we report effect estimates in the result on the original methylation values, such that effect estimates represent change in DNA methylation beta values for 1 g increase in birthweight.

### Gene expression analyses

We performed linear regression to test the associations between DNA methylation levels (*M*-values) of the birthweight-associated CpGs and placental gene expression of cis-genes (500 kb upstream and downstream of the CpGs). The methylation-expression association analyses were done in 75 individuals that had both DNA methylation and gene expression profiles from placental samples. For the significant methylation-expression associations (FDR < 0.05), we tested whether gene expression was associated with birthweight using Pearson correlation test.

### Genetic correlates of DNA methylation in placenta and blood

To examine whether genetic variants influence DNA methylation levels of the birthweight-associated CpGs, we explored the 15 CpGs in *cis*-methylation quantitative locus (*cis*-mQTL) resources. We queried the 15 CpGs using a list of previously published placental mQTLs [34, 35]. For the same query in blood, we used the mQTL database (http://www.mqtldb.org/) of mQTLs at serial time points across the life-course (pregnancy, birth, childhood, adolescence, and middle age) [36]. Furthermore, we assessed whether there are genetic variants within the proximity (± 500 kb) of the birthweight-associated CpGs by exploring the relative distances between our CpGs and birthweight GWAS loci in Warrington et al. [38]. The NHGRI-EBI GWAS catalog [37] was used to assess the relevance of the identified *cis*-mQTLs in birthweight and cardiometabolic diseases.

### Annotation, canonical pathway, and tissue enrichment analysis

Annotation of the nearest gene to the birthweight-associated CpGs was performed using the UCSC Genome Browser build hg19 (http://genome.ucsc.edu/). We performed genomic regulatory feature enrichment on the birthweight-associated CpGs using Fisher’s exact test. Regulatory regions tested for enrichment included CpG island locations, shelves, shores, transcription factor binding sites, and regulatory features from placenta-specific 15-chromatin state annotation (ChromHMM v1.9) from the ROADMAP Epigenomics Mapping Consortium [32].

Genes annotating the top 100 CpGs associated with birthweight were included in pathway analysis using the IPA tool (QIAGEN, Redwood City, CA, USA). Gene enrichment in canonical pathways was assessed using the right-tailed Fisher’s exact test. Pathways enriched at Benjamini-Hochberg corrected FDR *p* < 0.05 were considered statistically significant. In addition, we explored the top 100 CpGs for enrichment of overlap with tissue-specific or cell-type-specific DNase 1 hypersensitivity sites (DHS) using eFORGE v2.0 [33]. The set of 100 CpGs was entered as input in eFORGE and tested for enrichment for overlap with putative functional elements (DHS) compared to the consolidated ROADMAP epigenomics DHS. The enrichment analysis was performed in one thousand matched background sets, with each background set consisting 100 CpGs matched for gene and CpG island annotation. Enrichment above the 99.9th percentile (− log10 binomial *p* ≥ 3.38) was considered statistically significant as implemented in eFORGE.

### Evaluation of previously reported CpG sites associated with cardiometabolic traits

We used the EWAS Atlas database [39] to assemble CpGs in offspring tissues previously reported to be associated with maternal perinatal cardiometabolic traits (pre-pregnancy BMI, hypertensive disorders of pregnancy, and preeclampsia), and CpGs in adult/child blood previously reported to be associated with adult/child cardiometabolic traits (myocardial infarction, lipid traits, blood pressure-related traits, type 2 diabetes, and body mass index; see Additional Table 7). We queried our birthweight EWAS output to assess whether these previously reported CpGs were associated with birthweight at FDR < 0.05.

## Supplementary information

**Additional file 1: Additional Table 1.** Comparison of demographic characteristics of women with placental samples and women in the overall NICHD Fetal Growth Studies. **Additional Table 2.** Association between placental DNA methylation of 15 birthweight-associated CpG sites and gestational age at delivery. **Additional Table 3.** Regulatory region enrichment of the birthweight-associated CpG sites. A. regulatory features from placenta-specific 15-chromatin state annotation (ChromHMM v1.9). B. CpG island locations. C. Transcription factors. **Additional Table 4.** Enrichment for tissue-specific DNase 1 hypersensitivity sites (DHSs) of the top 100 CpGs associated with birthweight based on experimentally derived Functional element Overlap analysis of Regions from EWAS (eFORGE). **Additional Table 5.** Single nucleotide polymorphisms significantly associated with DNA methylation at three CpG sites (meQTLs) in the mQTL database (http://www.mqtldb.org/). **Additional Table 6.** Previously known associations between the birthweight-associated CpG sites and phenotypes. **Additional Table 7.** Published CpG sites associated with maternal cardiometabolic traits and adult cardiometabolic traits. **Additional Table 8.** CpG sites associated with maternal cardiometabolic traits and adult cardiometabolic traits in published studies as well as with birthweight in the present study (FDR <0.05).

**Additional file 2: Additional Figure 1.** Quantile-quantile (QQ) plot P-values for associations between CpG sites in placenta and birthweight.

## Data Availability

The placental DNA methylation, genotype, and gene expression data are available through dbGaP with accession number phs001717.v1.p1. The analytic codes for the current study are available upon request to the corresponding author.

## Abbreviations

BMI: Body mass index
CpG: Cytosine-phosphate-guanine genomic site
DHS: DNase 1 hypersensitivity site
eFORGE: Experimentally derived Functional element Overlap analysis of Regions from EWAS
eQTL: Expression quantitative trait locus
EWAS: Epigenome-wide association study
FDR: False discovery rate
GWAS: Genome-wide association study
IPA: Ingenuity pathway analysis
mQTL: Methylation quantitative trait locus
NHGRI: National Human Genome Research Institute
NICHD: *Eunice Kennedy Shriver* National Institutes of Child Health and Human Development
PC: Principal component
SNP: Single nucleotide polymorphism
SVA: Surrogate variable analysis
