# Supplementary figures and images for: DNA methylation loci in placenta associated with birthweight and expression of genes relevant for early development and adult diseases
Source: Clin Epigenetics. 2020 Jun 3;12:78. doi: 10.1186/s13148-020-00873-x (PMC7268466; doi:10.1186/s13148-020-00873-x)

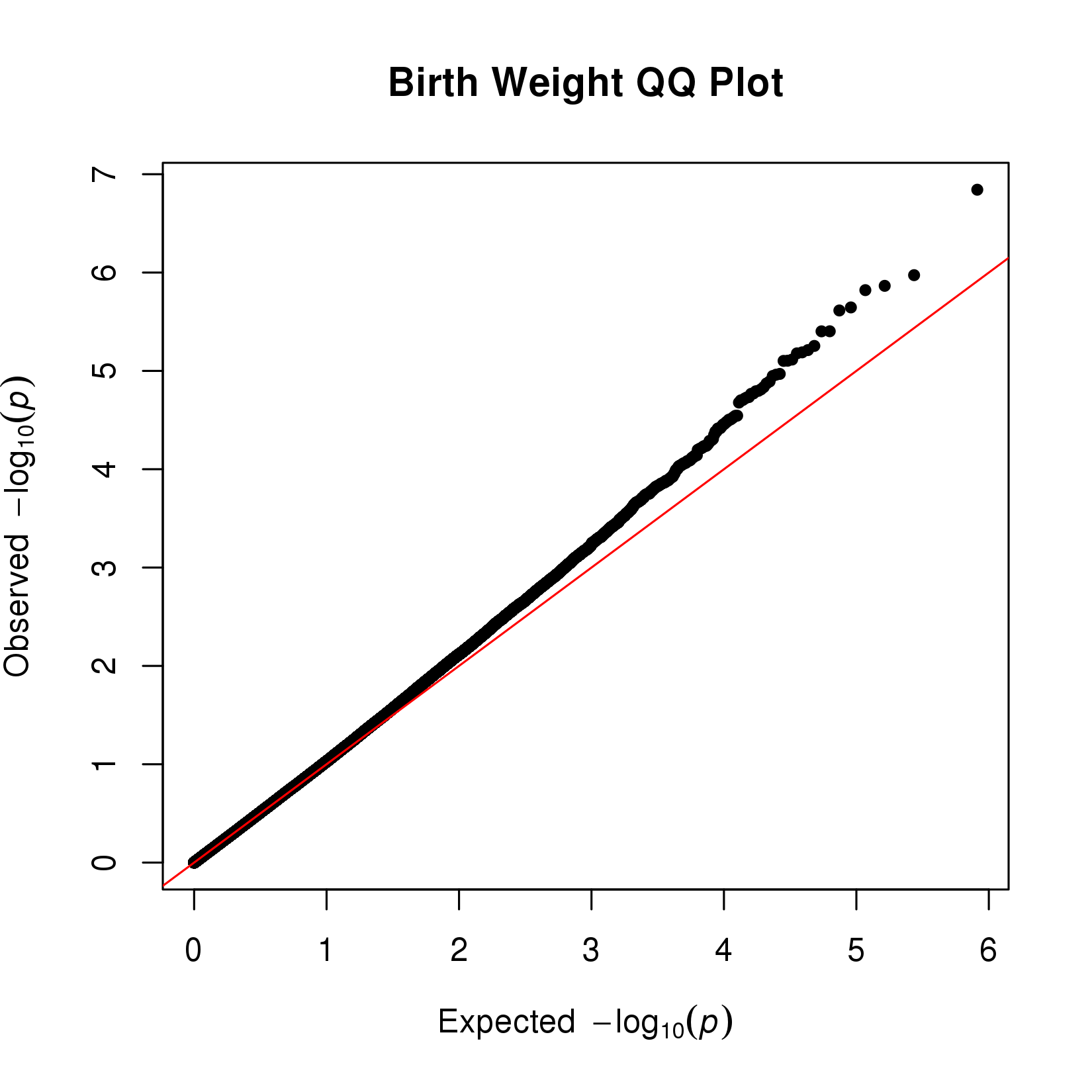

Supplement: Supplementary file 2 — Additional file 2: Additional Figure 1. Quantile-quantile (QQ) plot P-values for associations between CpG sites in placenta and birthweight. [file 13148_2020_873_MOESM2_ESM.png]
